# Second Harmonic Generation from Phase-Engineered Metasurfaces of Nanoprisms

**DOI:** 10.3390/mi11090848

**Published:** 2020-09-12

**Authors:** Kanta Mochizuki, Mako Sugiura, Hirofumi Yogo, Stefan Lundgaard, Jingwen Hu, Soon Hock Ng, Yoshiaki Nishijima, Saulius Juodkazis, Atsushi Sugita

**Affiliations:** 1 Department of Applied Chemistry and Biochemical Engineering, Shizuoka University, 3-5-1 Johoku, Hamamatsu, Shizuoka 432-8561, Japan; mochizuki.kanta.15@shizuoka.ac.jp (K.M.); maco_0213_8@yahoo.co.jp (M.S.); hirofumi_yogo@nidek.co.jp (H.Y.); 2 Optical Sciences Centre and ARC Training Centre in Surface Engineering for Advanced Materials (SEAM), School of Science, Swinburne University of Technology, Hawthorn, VIC 3122, Australia; slundgaard@swin.edu.au (S.L.); jhu@swin.edu.au (J.H.); soonhockng@swin.edu.au (S.H.N.); 3 Department of Physics, Electrical and Computer Engineering, Graduate School of Engineering, Yokohama National University, 79-5 Tokiwadai, Hodogaya-ku, Yokohama 240-8501, Japan; nishijima@ynu.ac.jp; 4 Institute of Advanced Sciences, Yokohama National University, 79-5 Tokiwadai, Hodogaya-ku, Yokohama 240-8501, Japan; 5 World Research Hub Initiative (WRHI), School of Materials and Chemical Technology, Tokyo Institute of Technology, 2-12-1, Ookayama, Meguro-ku, Tokyo 152-8550, Japan

**Keywords:** metasurfaces, second harmonic generation, phase control, finite difference time domain

## Abstract

Metasurfaces of gold (Au) nanoparticles on a SiO${}_{2}$-Si substrate were fabricated for the enhancement of second harmonic generation (SHG) using electron beam lithography and lift-off. Triangular Au nanoprisms which are non-centro-symmetric and support second-order nonlinearity were examined for SHG. The thickness of the SiO${}_{2}$ spacer is shown to be an effective parameter to tune for maximising SHG. Electrical field enhancement at the fundamental wavelength was shown to define the SHG intensity. Numerical modeling of light enhancement was verified by experimental measurements of SHG and reflectivity spectra at the normal incidence. At the plasmonic resonance, SHG is enhanced up to ∼3.5 × 10${}^{3}$ times for the optimised conditions.

## 1. Introduction

Energy up-conversion is important for a diverse range of fields, including—non-linear optics (NLO) and generation of higher laser harmonics, harvesting of long-wavelength, sub-bandgap energy light in solar cells, and photo-thermal excitation of neurons at the near-IR transparency window in tissue [1,2,3]. Creating efficient strategies for generation of second and higher harmonics of light using non-linear $\chi^{\left(2\right)}$ and $\chi^{\left(3\right)}$ responses of metasurfaces is a recent and active line of research [4,5,6,7,8,9]. The intense and confined surface plasmon enhanced optical fields are suitable for conducting the nonlinear wave mixing in the sub-diffraction spaces.

For conventional nonlinear wave mixing in the bulk NLO medium, the phase-matching conditions have to be satisfied. The group velocity of the harmonic wave is different from the fundamental because of the difference in the refractive index between them. The fundamental and harmonic waves become out of phase during the longer light-matter interaction length, resulting in the newly converted harmonic wave destructively overlapping with the previously converted wave. At the nanoscale, the phase matching conditions that are required for efficient energy transfer from the fundamental wavelength λ into higher harmonic λ/2 along the co-propagation direction are relaxed at the near-field, however, the efficiency of the second harmonic generation (SHG) is low. By using an array of nano-antennas on the surface of lithium niobate, it was demonstrated that the phase matching for SHG can be overcome in waveguides [10], however, high precision nanoscale fabrication is required. A simpler approach is when the lack of phase matching is compensated by the field enhancement using geometrical factors of nanoparticles. For example, an array of gold nano-bumps made by controlled ultra-short laser pulse fabrication showed up to ∼10${}^{2}$ times stronger SHG as compared with flat film of gold [11]. The light field enhancement by nano-bumps at the fundamental wavelength defined the enhancement of SHG.

Controlled laser melting on the nanoscale can be used to create nano-bumps on glass [12] for subsequent coating with plasmonic metals. Evidence of a strong light field enhancement by gold nanoparticles separated by nano-gaps was demonstrated by detection of usually weak two-photon excited photoluminescence from gold [13]. Randomly ordered nano-cavities in gold can achieve extraordinary transmission and light field enhancement [14]. By use of Mie resonance of AlGaAs-on-Insulator nano-cylinders, the SHG was shown to increase by 50 times in the zero-order diffracted beam [15]. Harnessing the 3D collective coupling between electric quadrupole and magnetic dipole modes of plasmonic nanoparticles, an enhancement and spectral control of SHG was predicted numerically [16]. Metasurfaces of lithium niobate nano-cylinders can reach $10^{-5}$ conversion efficiency into the second harmonic [17].

Recently, we showed that the phase control of light reflected and incident on a nanoparticle can enhance surface enhanced Raman scattering (SERS) [18,19]. This mechanism can provide an additional control for the phase sensitive SHG based on the π phase change upon reflection when light travels through the low-to-high refractive index boundary and the 0 phase change for traversing the high-to-low interface. This mechanism based on Fresnel coefficients is explored in this study together with a propagation phase control by thickness of glass layer.

It was shown recently that optically induced magnetization of gold nanoparticles due to the inverse Faraday effect can be harnessed for non-reciprocal ultra-fast optical rotation [20]. Also, nanoparticles of Ag formed by annealing of the implanted Ag${}^{+}$ into a Pr:CaF${}_{2}$ laser crystal host broadens and enhances spectral emission of Pr${}^{3+}$ required for a shorter laser pulse generation [21]. The use of nanoparticles and nanostructures in optical control of light enhancement, propagation direction, reflection and NLO effects continues to evolve and widen.

Here, we demonstrate that phase control of light incident and reflected from the layered SiO_2_-Si structure allows controlled SHG enhancement. We fabricate and characterise metasurfaces made of plasmonic nanoparticles with a controlled-thickness SiO_2_ spacer layer on top of a Si substrate. Numerical modeling by finite difference time domain (FDTD) was carried out to reveal characteristics of light field enhancement.

## 2. Experimental

### 2.1. Fabrication of Metasurfaces

**Figure 1:**
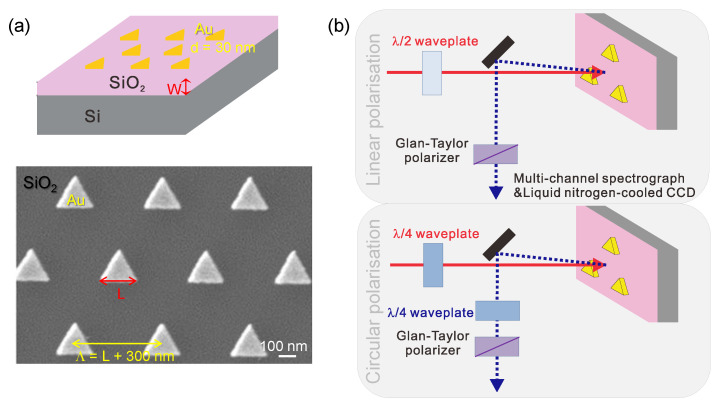
(**a**) Schematic of the sample (top) and an SEM image of the triangular Au nanoparticles (bottom). The spacer of SiO_2_ with width w=200,300 nm was deposited on the Si substrate to control the E-field enhancement at the plasmonic Au triangular nanoparticles. The pattern was triangular with period Λ=L+s where separation between nanoparticles was s=300 nm and the side-length of the triangle was L=(120−220) nm changed in steps of 20 nm. Thickness of Au nanoparticles made by electron beam lithography (EBL) and lift-off was d=30 nm. (**b**) Setup to detect second harmonic generation (SHG) from the metasurfaces under the linearly and circularly polarised excitation. The second harmonic light was analysed at $\approx 1^{\circ }$ reflection to the normal. This setup was used to maximise collection of the second harmonic. The excitation light source was Ti:sapphire fs-laser with the wavelength tunable from 730 to 920 nm.

Samples of plasmonic metasurfaces were prepared by standard electron beam lithography (EBL) and lift-off (Figure 1a). A 30-nm-thick gold film was sputtered on a triangular lattice pattern in ZEP520 resist. A thin film of 3 nm of Cr was deposited first for better adhesion of gold. Si(100) wafers were used as a substrate with w=200 and 300 nm thermally-oxidised SiO_2_. The oxidation was performed under a water-vapor atmosphere at 1000 ${}^{\circ }C$ with thickness determined by the hold time at temperature.

### 2.2. Characterisation of Metasurfaces

Extinction-total losses due to absorption and scattering- was measured using a fiber-coupled tungsten-halogen lamp (SLS201L/M, Thorlabs) for the white light source. For the femtosecond laser radiation we used a mode-locked Ti:sapphire oscillator (Tsunami, Spectra-Physics). The oscillation wavelength was tunable between 730 and 920 nm, and the pulse width and repetition rates were ∼100 fs and 75 MHz, respectively. Second harmonic generation (SHG) was measured under wavelength tunable fs-laser irradiation of metasurfaces at normal incidence. The SHG signals were detected in the back-reflection geometry. Linear polarisation of the incident light at λ=800 nm wavelength was set either with λ/2 or λ/4-plate (Figure 1b). The former was used for rotating the polarisation direction of the linearly-polarised light. The latter was used for converting the linearly-polarised light into the circularly-polarised light. Polarisation of SHG was interrogated with a Glan-Taylor polariser for the linearly-polarised excitation. The combination of the second λ/4-plate and the Glan-Taylor polariser was used for analyzing the polarisation state of the SHG waves converted from the circularly-polarised excitation [22] (Figure 1b).

The unconverted portion of the fundamental light propagated almost collinearly to the SHG waves. It was removed by a color glass filter (FGB37, Thorlabs, Newton, NJ, USA). The SHG signals were detected by liquid nitrogen-cooled CCD camera after being spectrally resolved by a multichannel spectrograph (SpectraPro SP-500, Princeton Instruments, Trenton, NJ, USA).

### 2.3. Numerical Modeling

**Figure 2:**
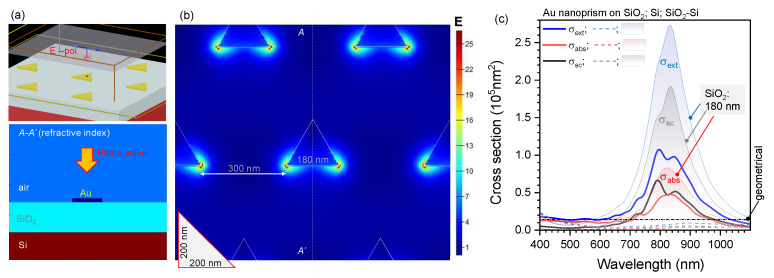
(**a**) 3D finite difference time domain (FDTD) setting for calculations under linearly polarised (along x-axis) E-field; plane wave illumination. Refractive index cross section (*A-A’*). (**b**) E-field $E/E_{0}$ cross section at the middle-plane of 30-nm-thick Au nano-particles (15 nm above SiO_2_). The incident field $|E_{0}|=1$. The maximum field cross section shown is at λ=825 nm as in the experiment, see text for discussion. (**c**) Absorption, scattering and extinction cross sections $\sigma_{ext}=\sigma_{abs}+\sigma_{sc}$ for the L=180 nm nanoprism on SiO${}_{2}$ (solid lines; refractive index n=1.4), Si (dashed-lines), and SiO${}_{2}$ (w=180 nm)-on-Si; optical properties of Si were taken from the material database of Lumerical. The FDTD calculations were carried out using total-field scattered-field (TFSF) light source. Geometrical cross-section corresponds to the footprint area of the nanoprism $S_{Au}=\frac{\sqrt{3}}{4}L^{2}\approx 0.1403\times 10^{5}$ nm${}^{2}$.

Numerical simulations of light field enhancement were carried out by finite difference time domain (FDTD) method using Lumerical FDTD Solutions. Permittivity of Si, SiO_2_, and Au were taken from the database included within the software. Periodic boundary conditions were used for the triangular lattice pattern under auto-optimised mesh size (Figure 2a,b).

Cross sections of absorption $\sigma_{abs}$, scattering $\sigma_{sc}$ and extinction (i.e., the total losses $\sigma_{ext}=\sigma_{abs}+\sigma_{sc}$) were calculated using total-field scattered-field light source (Figure 2c). Nanoprisms with side length of L=180 nm made on SiO_2_ or SiO_2_-on-Si showed strong scattering around 800 nm wavelength which was used in this study for SHG from such metasurfaces. At this nanoprism size, the scattering is stronger than absorbance which is also important for efficient SHG. Only a SiO_2_ spacer thickness of w=180 nm is shown in Figure 2c to illustrate the effect of markedly increased scattering. Nanoprisms on Si had red-shifted resonance and is outside the scope of this study. It is noteworthy, that light E-field enhancement is even stronger at the Au-Si interface as compared with Au-SiO_2_ and can be useful for sensor applications in the IR spectral range. These numerical estimates of light absorption and scattering by single nanoprisms was encouragement to embark on fabrication of arrays with different sized nanoprisms on reflective Si substrates with different SiO_2_ spacer thicknesses.

## 3. Results and Discussion

The second-order NLO responses of the metal nanoparticles are expressed by the surface integral of the local non-linear polarisations created on the metal surfaces [23]. The second-order NLO susceptibilities $\chi^{\left(2\right)}$ of the metal surfaces are predominantly determined by the surface effects [24,25,26]. The electric-dipole type selection rule is applied for expressing the non-linear wave conversions on the metal nanoparticles [27]. The geometries of the triangular nanoprisms are classified into the *D3h* point group, in which the system is invariant against mirror reflection to the yz-plane and 3-fold rotation around z-axis. In this case, there are only three non-zero non-linear tensor components $\chi_{yxx}^{\left(2\right)}$, $\chi_{yyy}^{\left(2\right)}$, and $\chi_{xxy}^{\left(2\right)}$ [28], where the x and y-directions are defined as the base and height directions of the triangular nanoprisms. These tensor components are related mutually as $\chi_{yyy}^{\left(2\right)}=-\chi_{yxx}^{\left(2\right)}=-1/2\chi_{xyx}^{\left(2\right)}=\chi_{0}^{\left(2\right)}$, and they are expressed with only one parameter $\chi_{0}^{\left(2\right)}$. The field distributions calculated by FDTD distributions were symmetric in the y-axis for both of the pump polarisation geometries and asymmetric in the x-axis. Thus, the SHG emissions along the y-axis was related to breaking symmetry in the x-directions of the electric field, hence, the non-linear tensor components $\chi_{yxx}^{\left(2\right)}$ and $\chi_{yyy}^{\left(2\right)}$ were responsible for the second-order non-linearities. Next, we tested experimentally the angular dependence of scattering at different excitation wavelengths and polarisation dependence at the maximum of the SHG at 800 nm for nanoprisms on glass samples.

### 3.1. Au Triangular Nanoprisms on Glass

**Figure 3:**
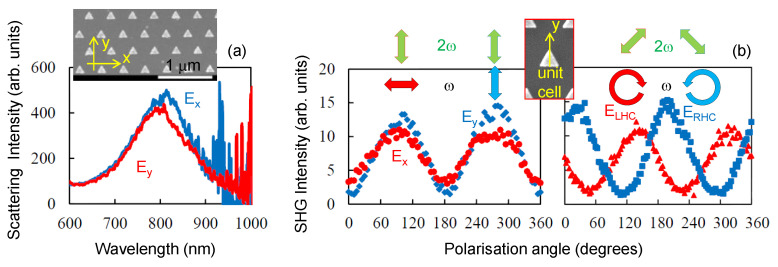
(**a**) Scattering spectrum of Au nanoprisms on glass for two polarisations in back-scattering geometry. The sizes of the nanoprism were: L=150 nm base of the equilateral triangle, 30 nm thickness, corner-to-corner separation was 250 nm. The prisms arranged two-dimensionally in a trigonal lattice (see SEM image in inset). (**b**) Polarisation-resolved SHG (2ω) at 800 nm (ω) excitation for different linear and the circular (left- and right-hand) polarisations of excitation in back-scattering/reflection geometry. SHG was y-polarised for different angles of orientation of the incident linearly polarised light (ω). SHG was linearly polarised at $\pm 45^{\circ }$ from y-axis for the LHC and RHC excitation (ω); see Figure 1b.

Figure 3 shows the scattering spectrum from the Au nanoprisms. The scattering spectra were probed by light with polarisations either parallel or perpendicular to the baselines of nanoprisms (x- and y-pol. respectively). The spectral shape of the scattering signal was independent on the polarisation direction of the probe light and peaked at 800 nm. The linear optical properties of the nanoprisms were isotropic. The y-polarized SHG signals were generated from both the *x*- and *y*-polarized excitation light (Figure 3b). The observed results are explained by the tensor forms above-mentioned. The non-linear polarisations are expressed as $\vec{P}\left(2\omega \right)=\left[\chi_{0}^{\left(2\right)}\cdot \left(E_{y}{\left(\omega \right)}^{2}-E_{x}{\left(\omega \right)}^{2}\right),-2\chi_{0}\cdot E_{x}\left(\omega \right)\cdot E_{y}\left(\omega \right)\right]$ against the fundamental light $\vec{E(\omega )}=\left(E_{x}\left(\omega \right),E_{y}\left(\omega \right)\right)$ by using the tensor components. Under the irradiation of the linear polarised excitation light, of which direction is θ with respect to the *y*-axis, or $\vec{E(\omega )}=\left(E_{0}\left(\omega \right)\cdot \sin\theta ,E_{0}\left(\omega \right)\cdot \cos\theta \right)$, the non-linear polarisation $\vec{P(2\omega )}=\chi_{0}\cdot E_{0}{\left(\omega \right)}^{2}\left(\sin\left(-2\theta \right),\cos\left(-2\theta \right)\right)$ is generated. The equation expresses the circles, and that the intensity of the non-linear polarisation is isotropic, independent of θ. The *x*- and *y*-polarised fundamental light corresponds to $\theta =90^{\circ }$ and $0^{\circ }$, respectively. In both cases, the polarisation is $\vec{P(2\omega )}=\chi_{0}^{\left(2\right)}\cdot E_{0}^{2}\left(\omega \right))\left(0,1\right)$ for $\theta =90^{\circ }$. Both the *x*- and *y*-polarized fundamental light should be converted into the y-polarized non-linear polarisation.

In addition, the maximum SHG intensities for the *x*-polarised excitation are almost comparable that of the *y*-polarized one (Figure 3b). The intensity of the nonlinear polarisation is $|\vec{P}\left(2\omega \right)|=\varepsilon_{0}\cdot {\vec{E}}_{0}^{2}\left(\omega \right)$ both at θ = 0${}^{\circ }$, and 90${}^{\circ }$, corresponding to x- and y-polarised pumps, respectively. Hence, the observation of $I_{SHG}\left(\theta =0^{\circ }\right)=I_{SHG}\left(\theta =90^{\circ }\right)$ makes sense from the viewpoint of the point symmetry of the Au nanoprism. This is also elucidated in the next section in terms of the field distribution calculated with the FDTD method.

According to the tensor form, the LHC excitation with $\vec{E}=\frac{E_{0}}{\sqrt{2}}\left(1,i\right)$, should be converted into the non-linear polarisation, $\vec{P}=i\cdot \chi^{\left(2\right)}\cdot E_{0}^{2}\left(1,-i\right)$, that is, the one with the RHC. Similarly, the RHC excitation light should be converted into the non-linear polarisation with the LHC. The SHG spectroscopy was performed for the nanoprisms with right- and left-handed circular (RHC, LHC) polarised excitation. The SHG intensities transmitted continuously through the λ/4-plate and the polariser was the highest at +45${}^{\circ }$ for the LHC excitation and −45${}^{\circ }$ for the RHC (Figure 3b). The rotation direction of the SHG waves had to be opposite to that of the fundamental. The polarisation state observed by λ/4-plate and the Glan-Taylor prism is consistent with the expectation imposed by the tensor form for the structures with the *D3h* symmetry [22].

Next, we investigate how SHG can be controlled by increasing light field enhancement at the excitation wavelength.

### 3.2. Au Triangular Nanoprisms on Si with SiO${}_{2}$ Spacer

Triangular nanoprisms with different side length from L=120 to 140 nm were fabricated on a strongly reflective Si substrate with two different SiO${}_{2}$ spacer thicknesses of w=200 nm and 300 nm (Figure 1).

**Figure 4:**
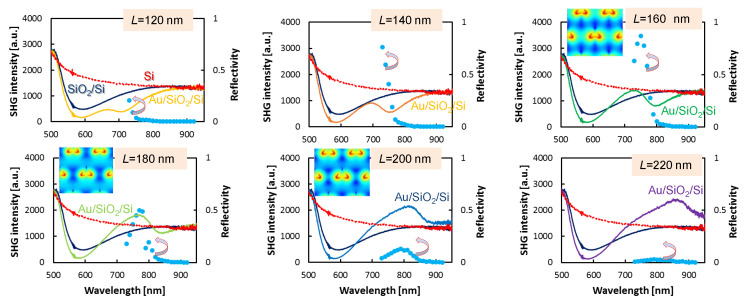
Plots showing experimentally measured SHG excitation spectra from metasurfaces (cyan dots, left-axis) of Au triangular nanoparticles on a SiO_2_/Si substrate with triangle side-lengths, L=120 nm to 220 nm. Reflectivity spectra R(λ) (right-axis) are shown for bare Si (red), Si with SiO_2_ (dark blue), and the metasurface for different size *L* nano-prisms (color coded). The SiO_2_ spacer width was the same w=300 nm (see Figure A1 for w=200 nm). Polarisation of the incident field was horizontal $E_{x}$. The insets show lg(E) maps of the calculation cell at the wavelength of maximum enhancement, which was at 824 nm for L=160,180 nm and at 871 nm for L=200 nm (see text for details).

**Figure A1:**
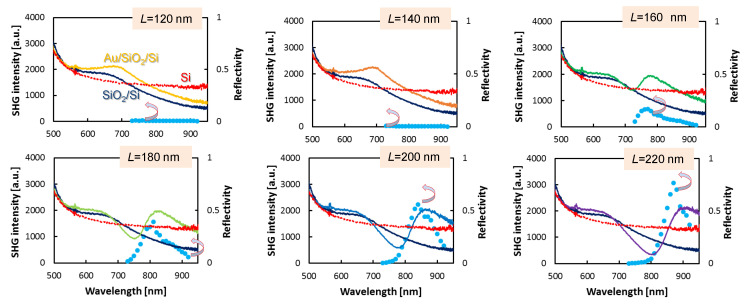
Plots showing experimentally measured SHG excitation spectra from metasurfaces (cyan dots, left-axis) of Au triangular nanoparticles on a SiO_2_/Si substrate with triangle side-lengths, L=120 nm to 220 nm. Reflectivity spectra R(λ) (right-axis) are shown for bare Si (red), Si with SiO_2_ (dark blue), and the metasurface for different size *L* nano-prisms (color coded). The SiO_2_ spacer width was the same w=200 nm. Polarisation of the incident field was horizontal $E_{x}$.

Figure 4 (also, see Figure A1) summarise reflectivity and SHG results from metasurfaces with different side length *L* of nanoprisms. The largest spectral sensitivity of SHG vs size of nanoprism *L* was observed for the thicker w=300 nm spacer (Figure 4). For thinner w=200 nm, the SHG from L=120 and 140 nm metasurfaces was measurable but at the level of tens-of-counts (Figure A1) and the strongest SHG was observed for L=220 nm. It is instructive to compare spectral SHG response with reflectivity spectrum. An increase of SHG was observed when the SiO_2_ spacer conferred anti-reflective properties to the surface (*R* smaller as compared with bare Si). The reflectivity of a metasurface with Au nanoprisms is defined by the geometry: period and size of nanoparticles. At peak reflectivity of the fundamental wavelength, the strongest SHG was observed.

FDTD simulations confirmed the main observations. Figure 2b shows the maximum SHG at λ=825 nm. For w=200 nm, SHG was enhanced for larger nanoprisms L≥180 nm (Figure A1). Larger triangles formed a larger unit cell of the triangular lattice, hence, the increase of SHG is affected as the ratio of metal area per unit cell, that is, $S_{Au}/S_{cell}\equiv \frac{L^{2}}{2\Lambda^{2}}$, where Λ=L+300 nm for the data shown in Figure 4 and Figure A1. For example, there is 32% more surface covered by gold for the larger particles L=200 nm as compared to the L=160 nm case. A more reflective surface contributes to the increase in SHG. Since the extinction of this particular pattern (specific L,w,Λ values) has a resonance related to material and geometry, there is an optimal *L* for the strongest SHG.

On the resonance at maximum reflectivity, SHG was enhanced more than $3\times 10^{3}$ times as compared with the non-resonant case (Figure 4). Also, the maximum of SHG was observed at shorter wavelengths. Obviously the effect of the spacer as a phase retarder for the light reflected from Si on a path to the nanoprism and positively interfering with incident and reflected light from the top of the SiO${}_{2}$ layer is an important factor. The exact spectral location of SHG maximum was also defined by the area where E-field enhancement is localised as can be better highlighted in logarithmic lg(E) scale as shown in three insets in Figure 4. The strongest SHG enhancement was observed from the L=160 nm sample where the largest surface of high E-field values was observed on gold nanoprisms. SHG has contributions from areas with E-field enhanced by different amounts. Due to non-linear $I^{2}\equiv E^{4}$ character of SHG, only the regions with the strongest enhancement contributes (red in the insets). The insets show very slight but recognisable differences in the enhancement pattern; where the maximum enhancement is localised with maximal E≈31 (enhancement) for L=160,180 nm and 29 for L=200 nm. The spectral positions of *E* maxima did not exactly match the experimental peak of SHG (Figure 4). The E-field enhancement, its area and localisation on nanoprisms, total reflectivity defined by the surface area of gold, and geometrical resonance of extinction all are interconnected and deserve subsequent study with a wider parameter space for *w* values. This particular issue, to determine which region on a nanoparticle with E-field enhancement contributes to the measured signal will be addressed in the study of surface enhanced Raman scattering (SERS; a nonlinear $\chi^{\left(3\right)}$-process) where molecules of different size experience different E-field enhancement in the nano-gaps and will be reported separately [29]. Although the Au nanoprisms were made on Si substrates without SiO${}_{2}$ spacer, the plasmonic responses were not observed in the present spectral window due to a high ∼3.7 refractive index of Si causing the localized surface plasmon resonance condition satisfied at much longer wavelengths.

**Figure 5:**
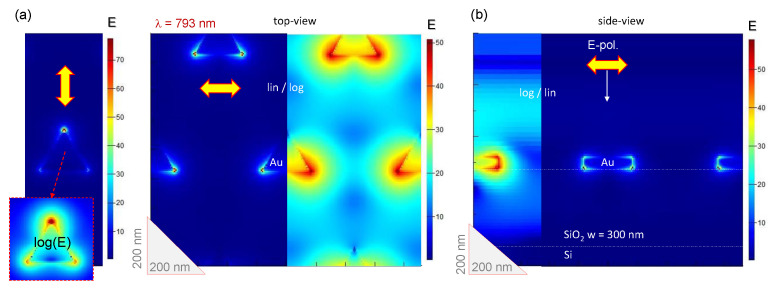
(**a**) Top view FDTD calculations at the maximum E-field enhancement for L=180 nm and w=300 nm (see Figure 4) for both $E_{y}$ and $E_{x}$ polarisations. The top-view monitor is at the air-SiO_2_ interface and the side-view monitor crosses the side of triangle and vertexes with the highest field enhancement. The E-field scale bars are linear; polarisation of incident field was horizontal $E_{x}$. Larger enhancement for $E_{y}$ orientation as compared with $E_{x}$ is manifested in corresponding scaling of SHG (See Figure 3). Calculations for the two $E_{x,y}\left(\omega \right)$ fields were carried out for the same unit cell. (**b**) Side view cross section for the $E_{x}$ excitation.

The maximum of SHG had well defined optimal conditions which corresponded to L=160 nm and w=300 nm. FDTD calculations confirmed the strongest light enhancement occurring at the tips of nanoprisms observed at the experimentally determined maximum of SHG enhancement. Figure 5a shows the light field enhancement close to the peak wavelength of SHG for L=180 nm. For the $E_{x}$-polarized excitation, the SHG conversion occurred around two tips, and the SHG intensity is $I_{SHG,x-pol}\;\eta_{x}^{2}=50^{2}\times 2=5\times 10^{3}$. For the $E_{y}$-polarized excitation, it occurred only at one tip and the SHG intensity is $I_{SHG,y-pol}\;\eta_{y}^{2}=70^{2}=4.9\times 10^{3}$. Thus, the relation of $I_{SHG,x-pol}∼I_{SHG,y-pol}$ is satisfied, and it is in a good agreement with the expectation derived from the tensor forms of the D3h point symmetry. The enhancement of the E-field of more than 50 times for $E_{x}$ polarisation of excitation and over 70 times for $E_{y}$ was observed and was located at the SiO_2_-Au interface. It is noteworthy that the absolute values of enhancement obtained by FDTD should not be considered due to ideal geometrical structures and interfaces being different due to fabrication tolerances [30,31,32]. The difference in SHG emission for the $E_{x}$ and $E_{y}$ (Figure 3b) followed the scaling of the field enhancement at the Au-SiO_2_ interface: the 2ω emission was stronger under $E_{y}$ excitation as compared with that at $E_{x}$.

The side-view FDTD cross section (Figure 5) reveals that some of incident light is scattered at larger angles from the direction of propagation at the edges/corners of the nanoprisms. This facilitates light trapping in SiO_2_ (between air and Si) which contributes to light enhancement at neighbouring nanoprisms.

**Figure A2:**
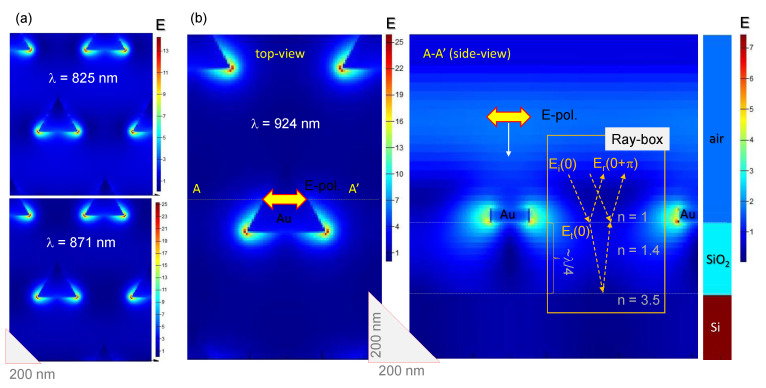
FDTD simulations for the L=220 nm w=200 nm case. (**a**) E-field enhancement at λ=825 nm (see Figure 2b for comparison) and 871 nm which are close-to-maximum. (**b**) Top- and side views of E-field enhancement for a linearly polarised plane wave. Refractive index cross-section is shown on right-side to distinguish the lateral cross-sections. Note, the cross-section *A-A’* is made though the center of triangle and not at the largest intensity vertexes. Incident light has $E_{x}$ polarisation. Inset “ray-box” shows schematically the phase change upon reflection from interfaces for the incident, transmitted and reflected E-fields $E_{i,t,r}$ in ray optics presentation [18]. In addition to the Fresnel coefficient defined phase changes, a propagation phase is adding up and amounts to π for traversing a λ/4 thickness twice upon back-reflection from Si.

The maximum of SHG is red-shifted for larger nano-triangles. This tendency was confirmed by FDTD simulations (Figure A2). The maximum of E-field enhancement was observed at 924 nm wavelength (L=220 nm) as compared with 825 nm for the L=180 nm. The pattern of E-field enhancement was qualitatively the same, the vertexes of equal-side triangles which are aligned to the polarisation of incident plane wave are enhanced. The peak enhancement up to E=30 times was observed at the maximum (incident field E=1). Side-view of E-field distribution shows even stronger localization at the SiO_2_-air-Au point (note, the lateral cross sections are shown at 15 nm above the interface at the middle thickness of Au nanoparticle). These locations of largest E-field locatization at the interface are locations for SHG. From the side-view image it is also clear that some light was deposited into the SiO_2_ spacer which also facilitates field enhancement at the neighbouring nanoparticles.

**Figure 6:**
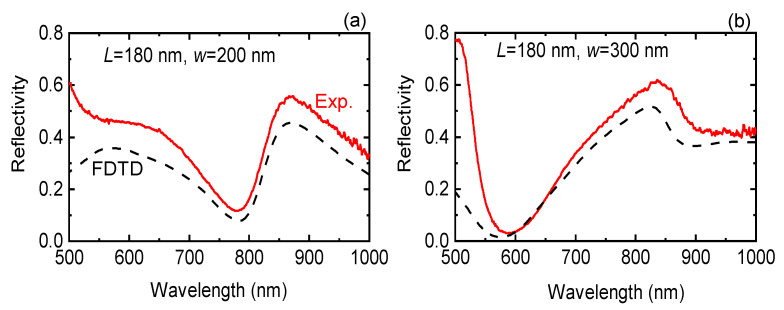
Experimental (Exp; red) and calculated (FDTD; dashed) reflectivity spectra of Au nanoprisms with L=180 nm side length. The thicknesses of the SiO_2_ spacer were (**a**) w=200 nm and (**b**) 300 nm.

Figure 6 shows direct comparison between experimentally measured reflectivity *R* together with FDTD numerical results for the two tested spacer thicknesses. Maximum of *R* was a good predictor for the most efficient SHG and a good match between theoretical estimates and experimentally measured *R* values was observed at the peak of SHG. It could be envisaged that by using different 2D and 3D nanofabrication techniques including direct laser writing [33,34,35,36] it should be possible to inscribe non-centro-symmetric patterns into the interface or fill by NLO polymers rendering such meta-surfaces/materials as efficient SHG materials [37]. The use of reflective plasmonic non-centro-symmetric patterns are very promising for nanoscale engineering of SHG [38,39]. The presented triangular symmetric *D3h* pattern of nanoprisms can be used to enhance SHG from 2D materials of the same symmetry, for example, WSe_2_, which showed SHG from monolayered flakes [40]. Also, photo and thermally induced material re-organisation can be used for breaking usually random orientation and symmetry of polymers to make them active for SHG [41]. Use of anisotropic bio-polymers such as silk [42], nanocellulose [43] and their polymer composites is another way to make host materials anisotropic for the light-matter interaction required for efficient SHG. Light localisation on nano-structured surfaces provides strong light gradients required for optical trapping/binding [44,45,46], which is useful for surface assisted light enhancement in sensing and fabrication [34,47,48,49,50], while generation of second harmonic at the nanoscale features could be explored for their contribution to the biocidal conditions [51,52,53].

## 4. Conclusions and Outlook

It is demonstrated that SHG from non-centro-symmetric triangular nanoprisms can be enhanced using a SiO${}_{2}$ spacer between the nanoprisms on a Si substrate. Experimental results proved that the Au nanoprisms are well suited for harnessing second-order non-linearities at normal incidence conditions in the nanoscale. The polarisation dependence of SHG showed that at the linearly polarised fundamental wave the SHG was always y-polarised, independent of the polarisation of the excitation light. For the circularly polarised excitation, the SHG was also circularly polarised with the handedness opposite to the excitation light as expected from the *D3h* symmetry.

By optimising the thickness of the SiO${}_{2}$ spacer it is possible to maximise SHG generation by several orders of magnitude. It is expected that this method will allow achievement of high yield SHG from films of non-linear optical (NLO) materials placed on metasurfaces—$I\left(2\omega \right)\propto |\chi_{meta}^{\left(2\right)}+\chi_{NLO}^{\left(2\right)}\times d_{NLO}{|}^{2}I^{2}\left(\omega \right)$ , where I(ω) is light intensity at the fundamental wavelength (λ=2πc/ω) which is locally enhanced at the nanoscale on the nanoprisms, $d_{NLO}$ is the thickness of the non-linear optical material which is expected to be thin for best harvesting of the local field enhancement. Metasurfaces of nanoparticles are expected to be able to withstand higher light intensities without degradation and has to be investigated next. As polymers enters the second century of their development [54], a combination of new polymers with augmented functionalities and metasurfaces will bring new science and applications.

## Appendix A. Reflection and SHG Spectra for *w* = 200 nm SiO_2_ on Si

Experimental spectra of reflectivity and SHG are overlayed for the metasurfaces with the side length of the nanoprism ranging from L=120 nm to 220 nm for the SiO${}_{2}$ spaced w=200 nm in Figure A1 (see main text for w=300 nm in Figure 4).

Figure A2 presents summary a of FDTD modeling of the light field enhancement for the w=200 nm SiO${}_{2}$ spacer at several wavelengths. The inset “ray-box” shows schematics of interference taking place on the front surface as addition of the SiO_2_-reflected and Si-reflected rays. Each of them experience π phase shift due to reflectance from the medium with a higher refractive index. The Si-reflected ray has additional propagation phase traversing the SiO_2_ spacer twice. When the spacer is close to the λ/4 condition, a constructive E-field addition takes place on the air-SiO_2_ surface (where Au nanoprisms are positioned). The actual field values depend on the Fresnel coefficients, which are, in turn, incidence angle dependent. This interference and phase matching is the physical reason for the increased SHG efficiency with the optimised thickness of SiO_2_ spacer around w=300 m [18]. Such description is strictly valid for the optical far-field representation of reflection and the actual near-field conditions where diffraction from the Au nanoprisms is taking place is accounted for in the FDTD simulations. More systematic studies are required for the dependence of SHG from the spacer thickness *w*. Here, only two thicknesses w=200 nm and 300 nm were tested at normal incidence. Angle dependent SHG has to be measured and more information on the light trapped in the waveguiding mode could be obtained.
